# Human Embryonic Stem Cells: A Model for the Study of Neural Development and Neurological Diseases

**DOI:** 10.1155/2016/2958210

**Published:** 2016-04-28

**Authors:** Piya Prajumwongs, Oratai Weeranantanapan, Thiranut Jaroonwitchawan, Parinya Noisa

**Affiliations:** ^1^School of Biotechnology, Institute of Agricultural Technology, Suranaree University of Technology, Nakhon Ratchasima 30000, Thailand; ^2^School of Anatomy, Institute of Science, Suranaree University of Technology, Nakhon Ratchasima 30000, Thailand

## Abstract

Although the mechanism of neurogenesis has been well documented in other organisms, there might be fundamental differences between human and those species referring to species-specific context. Based on principles learned from other systems, it is found that the signaling pathways required for neural induction and specification of human embryonic stem cells (hESCs) recapitulated those in the early embryo development* in vivo* at certain degree. This underscores the usefulness of hESCs in understanding early human neural development and reinforces the need to integrate the principles of developmental biology and hESC biology for an efficient neural differentiation.

## 1. Introduction

Development of the vertebrate central nervous system (CNS) is one of the earliest events in embryonic germ layer induction, and it has long been thought of as a step following the formation of the embryonic ectoderm [1]. This development involves multiple steps, beginning with the induction of neuroepithelium from the embryonic ectoderm and completing with the patterning of different parts of the brain. The CNS is a complex tissue, in terms of both the number of cells and the variety of cell types. In addition, billions of neurons have to interact in a very precise manner in order to form functional neuronal networks. The CNS is formed over time during embryogenesis and is rapidly converted from simple neural plate to a brain and spinal cord. To form a many different types of neurons and glial cells in the adult CNS, embryonic cells have to proliferate and differentiate in a strictly controlled manner, and during the last few years rapid progress has been made in understanding the molecular mechanisms underlying the initiation, proliferation, and differentiation of the CNS [2]. Mice, chicken, and zebrafish have long been considered model organisms for the study of vertebrate development. Studies of these organisms have provided details into the molecular mechanisms underlying embryonic development and are beginning to suggest potential pathophysiological mechanisms of some important development/congenital abnormalities in humans. However, in the ultimate quest to understand the mechanisms of human development with the goal of preventing and treating developmental defects in humans, these studies fall short. Understanding molecular interactions underlying human development is limited by the availability of human embryos and inadequate amount of stage-specific and cell type-specific materials. These problems may now be solved by the uses of human embryonic stem cells.

## 2. The Properties of Human Embryonic Stem Cells

Human pluripotent stem cells (hPSCs), here including human embryonic stem cells (hESCs) and human induced pluripotent stem cells (iPSCs), are capable of expanding indefinitely and differentiating into all human germ layers both* in vitro* and* in vivo *[3, 4]. During embryonic development, pluripotent stem cells are present only transiently and quickly differentiate into various somatic cells through developmental process [5]. However, it is possible to isolate* ex vivo* pluripotent mouse and human embryonic stem cells from the inner cell mass of blastocyst embryos and maintain them in laboratory. Human embryonic stem cells (hESCs) have the ability to renew and maintain their developmental potential to contribute to derivatives of all three germ layers, even after prolonged undifferentiated proliferation and/or clonal derivation [6]. In addition, hESCs can give rise to extraembryonic lineage, including trophectoderm and primitive ectodermal-like cells [4, 7]. Interestingly, hESCs are capable of expressing high level of telomerase, alkaline phosphatase, and key transcription factors that were also identified as being important in the maintenance of the inner cell mass pluripotency [8, 9]. These factors include the POU-family transcription factor OCT4, a homeodomain DNA-binding protein NANOG, and the SOX-family transcription factor SOX2. The embryonic markers defined by the antibodies SSEA-3 and SSEA4 are expressed by hESCs as well as the cell surface proteoglycan recognized by several monoclonal antibodies, including TRA-1-60 and TRA-1-81 [10]. The success derivation of hESCs provides a unique opportunity to study early human development and is believed to hold a great promise for biopharma and regenerative medicine [11, 12]. It is noted that the differentiation of hESCs in culture follows the hierarchical set of signals that regulate embryonic development in the generation of the germ layers and specific cell types at certain degree [13, 14]. Moreover, due to the difficulty of access to early human embryos and inadequate amount of stage-specific and cell type-specific materials, hESCs seem to provide a valid model to understand complex signaling interactions occurring in human embryos. In particular, the ability of hESCs to differentiate into defined neural lineages, neurons, astrocytes, and oligodendrocytes, is fundamental to study the sequence of events that take place during early neurodevelopment [14, 15]. Altogether, hESCs are a suitable and valid system to address the significant roles of the signaling pathways involved in neural lineage commitment and, ultimately, to model pathology of neurological disorders.

## 3. The Promising Applications of Human Embryonic Stem Cells

Although the success in establishing hESCs raised numerous ethical concerns, involving the development, usage, and destruction of human embryos, hESCs provide an alternative useful cell source for several potential applications in both basic science and medical treatment. To direct hESC differentiation* in vitro* along chosen pathways would allow for the investigation of early human development events, including regulatory signals for cell commitment and morphogenesis. Additionally, the cells could also be used for the screening of candidate drugs and carcinogenic or toxic compounds that cannot be analyzed in human embryos due to ethical constrains. However, investigations into the potential utility of hESCs in treating human diseases are at an infant stage because there are several issues needed to be taken into account, that is, efficiency, safety, and functionality [12].

The most urgent problem today in regenerative medicine is the lack of suitable donor organs and tissues. The pluripotent developmental potential of hESCs and the success of transplanting their differentiated derivatives into animal disease models reinforce the promising application of this cell type. This evidence has proofed the principle of using hESC-derived specific cells as a regenerative cell source for transplantation therapies of human diseases [16, 17]. One of the key issues causing hESCs technology to be useful for cell and tissue therapy in humans is the histocompatibility between graft and host. Recent data support the concept that hESCs and their differentiated derivatives possess immune privileged properties [18], suggesting that cells derived from hESCs may provide a potential tool for induction of immunotolerance [19]. On the basis of maternofetal immunotolerance observed during pregnancy along with the aforementioned immune privileged properties that ESCs share, the question of whether hESCs and their progeny can be considered potential vectors for tolerance induction in allogeneic recipients needs to become an area of active investigations [20, 21].

In another scenario for which the term “personalized pluripotent stem cells” has been coined, people could use their own somatic cells to be reprogrammed back to the pluripotent stem cell state. The feasibility of reprogramming was first demonstrated by somatic cell nuclear transfer (SCNT) or cloning. Somatic cells of patients are fused with enucleated oocytes; thereafter, hESCs could be established in culture and be induced to* in vitro* differentiation to provide patient-specific cells and tissues [22]. However, the reprogramming of somatic nucleus in an oocyte is still inefficient process. In addition, to access a source of human oocytes is not only a rare opportunity, but also an ethical concern worldwide [23]. As an alternative to reprogramming by SCNT, adult human fibroblasts can be directly reprogrammed into a state that is similar to hESCs by expression of only four factors, OCT4, KLF4, SOX2, and c-Myc [3], and term such reprogrammed cells as “induced pluripotent stem cells” or iPSCs. Nevertheless, techniques of reprogramming somatic cells are necessary to be nonviral, nononcogenic, and nongenetic modification before iPSCs can be used for treatment of human patients [24].

On the other hand, hESCs and their differentiated derivatives can be used in screening assays for the development of new potential pharmaceuticals and toxic as well as mutagenic compounds. While primary cell cultures or established cell lines are commonly used for both purposes, hESCs offer several advantages. hESCs have the ability to differentiate and efficiently produce unlimited numbers of cells representative of the three germ layers of embryos. The developmental equivalence of hESC-derived populations provides a more rigorous system for evaluating the teratogenic and embryotoxic effects of a substance, in addition to general mutagenic and cytotoxic effects [25]. A protocol based on hESCs differentiation has been established and validated for use in toxicity testing [26]. Additionally, genetic modification enables the tailoring of hESC lines for specific purposes. For example, specific genes can be altered to increase sensitivity to mutagens or drugs [27, 28], or tissue-specific reporter genes can be introduced to detect changes in gene expression induced by toxic chemicals or therapeutic agents [29].

Finally, understanding mammalian embryogenesis through analysis of the early embryo is complicated by a number of factors, including size, availability, and the complexity of the embryo and uterine environment. Since hESCs are precursors to all embryonic lineages, these cells allow tracing the history from the root to individual branches of the cell lineage tree in a simplified and controllable culture environment. System for differentiation of hESCs* in vitro* provides experimental models that can be used to augment* in vitro* studies of* in vivo* mammalian embryogenesis, promoting a greater understanding of genes and signaling pathways regulating developmental decisions. One concern is that cell culture does not have a complex cell and tissue interactions that are critical to embryonic induction at distinct developmental stages. These cellular interactions, however, can be largely recreated in culture in the future with combination of tissue engineering in order to reflect the* in vivo* environment, allowing the better system to study embryogenesis.

## 4. Neural Differentiation of Human Embryonic Stem Cells

Growing evidence from animal experiments has shown that the formation of the nervous system can be induced by signals that emanate from a region of the embryo known as the “organizer” which secretes several molecules containing a direct neural activity, including noggin, chordin, and follistatin. These molecules act as central inhibitors of the bone morphogenetic protein (BMP) signaling pathway, a conserved inhibitory mechanism to neurogenesis from arthropods to vertebrates [30]. BMP antagonism has been recognized as the key and initiating process in neural induction and neuroepithelial specification and this is believed to occur as* a default pathway* [31, 32]. Based on this fundamental, hESCs have been efficiently induced to neural progenitor cells by applying the BMP inhibitor, noggin, into the culture system [33, 34]. Nevertheless, other findings challenge this model and suggest that some additional factors, including fibroblast growth factors (FGFs) and Notch, also participate in neural induction process. Aberration of FGF signaling diminished neural induction process from mouse ESCs [35], and the defect of FGF signaling was shown to have interconnection with BMP pathway to prevent neural differentiation of stem cells [30]. Similarly, Notch signaling plays an important function for neural fate entry of ESCs [36]. Constitutive activation of Notch signaling in mouse ESCs does not alter their phenotypes but promotes neural differentiation upon withdrawal self-renewal stimuli. In contrast, inhibition of Notch signaling suppresses the neural fate commitment. However, it was suggested that Notch signaling which induces neural differentiation requires parallel signaling through FGF pathway [37]. For this reason, a balanced view of neural induction process most likely demands incorporating both instructive and inhibitory signals.

Several strategies have been employed to achieve* in vitro* neural differentiation from hESCs, aiming at producing region specific neural progenitor cells or mature neuron/glial subtypes [38–40]. This was primarily accomplished by cell aggregation or embryoid body (EB) formation in neural induction medium and highly purified populations of neural progenitor cells could be further isolated and cultured [41, 42]. These neural progenitor cells could be expanded for over 25 population doublings as neurospheres in suspension culture. The neurospheres express markers of neuroectoderm, including Nestin, polysialylated (PSA) N-CAM, Musashi1, and PAX6 [41]. Importantly, the neural progenitor cells can differentiate into all derivatives of the nervous system, which are neurons, astrocytes, and oligodendrocytes. Importantly, EB-based protocol could induce specific neuronal subtypes, for instance, forebrain cholinergic neurons, which showed mature and functional electrophysiological profile [43]. However, since hESCs are pluripotent, neural differentiation via EB culture contains several limitations. Firstly, because of the cluster nature of EB, it is difficult to visualize the continual change of cell morphology in response to treatment. Next, the efficiency of neural conversion by EB formation is limited and neural lineage selection is necessary to ensure the enrichment of neural progenitor population. Besides, the structure of hESC aggregates prevents uniformly distribution of supplemented morphogens or growth factors. A high concentration of morphogens or growth factors is needed in order for the factors to diffuse inside the cell aggregates [44]. Therefore, cells on the surface of EB and those inside the aggregates will encounter a varied gradient of morphogens. And, due to this reason, a wide range of cell fates or cells at distinct developmental stages are derived from neurospheres. To overcome the limitations of EB protocol, a simpler way to reconstitute neural differentiation and achieve high efficiency of neural progenitor cell production is based on monolayer differentiation system of hESCs. It was noted that when applying similar monolayer differentiation system used for directing mouse ESCs to neural fate, hESCs became a large proportion of nonneural lineage cells. This mainly results from the highly active BMP signaling pathway in hESCs [7]. Thereafter, the success approach, showing to induce efficient neural conversion of hESCs, is by directly inhibiting the BMP/SMAD signaling [33, 34]. Supplementation of hESCs with noggin, a BMP antagonist, in neural inducing medium generated a highly pure and morphologically distinct population of cells that expressed several neural progenitor cell markers, including PAX6, Musashi1, and SOX2, without the detection of mesodermal and endodermal lineage markers [33]. To reinforce the purity of desired neural progenitor cells, the use of neural specific regulatory element to control expression of fluorescent protein is a powerful alternative tool for efficient identifying and isolating of hESC-derived neural progenitor cells by fluorescence cell sorting technique [45].

## 5. Human Embryonic Stem Cells as a Model of Human Neural Development

**Figure 1:**
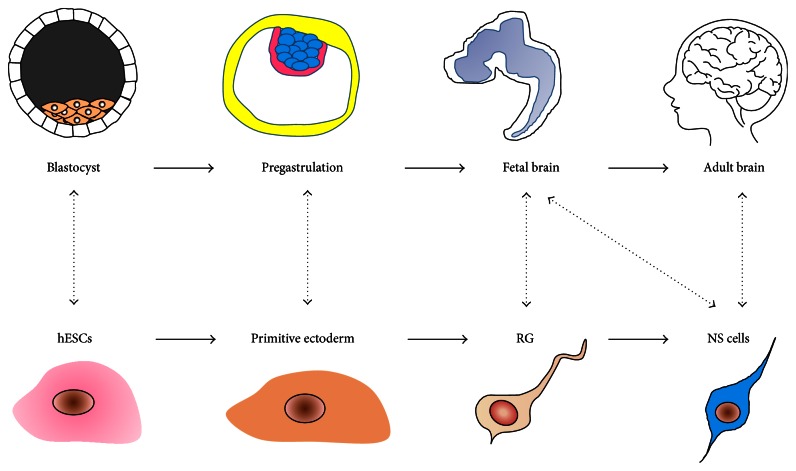
Developmental links between the different stages of neural derivatives of hESCs and their* in vivo* counterparts. Neural derivatives exhibit several similar characteristics to* in vivo* counterparts. The corresponding* in vivo* developmental stages are indicated and matched with the* in vitro* populations.

The embryonic origin of the brain is ectoderm. During neurulation, the neural plate folds over on itself and becomes the neural tube. Consequently, the forebrain, midbrain, and hindbrain of the central nervous system are patterned and formed. Cell fate determination within the developing brain is controlled by signaling molecules, secreted by neighboring tissues. The development of animal models for neurological disorders is challenging and often questioned whether it fully recapitulates the human phenotypes. hESCs offer an alternative approach, because neural cells differentiated* in vitro* from hESCs display several properties equivalent to the developing embryonic brain [46]. As mentioned above, at least two systems have been well developed to explore human neurodevelopment from hESCs, which are cell aggregation and cell adherent culture system [42, 47]. Prominently, directed differentiation of hESCs in an adherent system shows remarkable similarity between* in vitro* differentiation and* in vivo* neuroectodermal development (Figure 1). The morphology of hESCs converts to columnar neuroepithelium after 7–10 days of the differentiation, which further develop into neural tube-like rosette structure at days 14–17 of the differentiation [42]. Because hESCs are generally derived from 5-6 day-old blastocyst embryos, generation of columnar neuroepithelium at day 10 of the differentiation, and formation of neural rosettes at days 14–17 correspond gastrulation phase at the start of the third week, later, and the establishment of the neural tube at the end of the third gestation week of a human embryo [48], respectively. After the completion of the neural plate development* in vivo*, the generation of neural tube will successively begin but will not take place homogeneously and synchronize throughout the developing neural tube. Instead, the neural tube is patterned to dorsoventral and rostrocaudal domains in order to set a grid-like structure of positional cues along its axes [49]. This emphasizes the critical need to establish positional information that could efficiently facilitate the generation of particular subtypes of neuron and glia cells* in vitro* from hESCs. To simulate the positional instruction in a laboratory culture, morphogens or growth factors that affect dorsoventral and rostrocaudal fate choices could be applied at the same time or in a sequential manner. By applying FGF8, which is known to influence mid-hindbrain neuron phenotype, and sonic hedgehog (SHH), a ventralizing factor, further prime hESC-derived neural progenitor cells into midbrain dopaminergic neurons [50]. Absence of these positional factors in the* in vitro* differentiation leads to the production of heterogeneous neuronal subtypes. This suggests that the supplementation of a specific set of morphogens at a specific time point is essential to pattern neural progenitor cells into a desired neuronal subtype [15, 51].

Formation of “neural rosette structure” is a morphological hallmark of an* in vitro* differentiation of hESCs to neural lineage, which mimic the* in vivo* structure of developing neural tube [48]. The culture of hESCs in chemically defined medium with BMP inhibitor, noggin, resulted in the generation of PAX6^+^/SOX1^−^ neural rosettes and succeeding supplementation of FGF2 induced PAX6^+^/SOX1^+^ neural progenitor cells [52]. Rosette-forming neural progenitor cells that express forebrain markers, such as Forse1, have presented the broadest differentiation potential, compared to other neural progenitor cell populations [53]. These cells can be propagated in the presence of FGF2 and retained high Forse1 expression level, although FGF2 was recognized as caudalizing factor of neural progenitor cells [39]. Besides, the cells in neural rosettes are able to multiply by symmetric cell division and are capable of differentiating into cell types of both anterior-posterior, central-peripheral neuronal subtypes of the nervous system and are stable in a long term culture by stimulating SHH and Notch signaling pathways [53]. hESC-derived neurons can also be used to study synaptogenesis when plated onto specific feeder cells [54]. In addition to functional neurons, hESCs-derived neural progenitor cells are also able to produce astrocytes and oligodendrocytes either under basal conditions or instructive culture system, which is medium supplemented with ciliary neurotrophic factor or platelet-derived growth factor (PDGF) [55]. It is accepted that during early neurodevelopment, glial cells, including astrocytes and oligodendrocytes, are presented after the emergence of most neuronal cell types [56]. The similar scenario of neurogenesis to gliogenesis transition is conserved when explanted neuroectodermal cells are cultured or hESCs are differentiated along the neural lineage [14, 57]. Transcriptomics profile during neural differentiation of hESCs reveals distinct molecular features of multistage neural derivatives. The information obtained from this study might reflect mechanisms underlying brain development of human embryos [14]. The temporal changes of neuronal and glial differentiation of hESC-derived neural progenitor cells noteworthy reminiscent the timeframe observed from samples of embryonic tissues. This is suggested that the intrinsic program governing neuronal and glial lineage development is retained for hESC differentiation. Differentiated astrocytes, a robust derivative of hESC-derived neural progenitor cells, commonly express specific astroglial markers, including glial fibrillary acidic protein (GFAP) and S100*β*; however, oligodendrocytes are considered as a rare population obtained from hESC-derived neural progenitor cells [42]. It has been demonstrated that OlLIG2-positive neural progenitor cells can be readily obtained from hESCs in response to the treatment of SHH and RA [58]. These OLIG2-positive progenitor cells generate majorly motor neurons during neurogenesis; however, OLIG2-positive progenitor cells remain after neurogenic period and become mature oligodendrocytes. This suggests that the OLIG2-positive neural progenitor cells can give rise to oligodendrocytes and highlights the importance of OLIG2 in oligodendrocyte development* in vivo* [59].

## 6. The Approach to Model Neurodevelopmental Disorders by Human Embryonic Stem Cells

Neurodevelopmental disorders are caused by the impairment of the central nervous system during embryonic and early postnatal life. Early onset of neurodevelopmental disorders that are caused by genetic mutations could be probed by hESCs. This employs the advancement of preimplantation genetic diagnosis (PGD) during* in vitro* fertilization. Embryos diagnosed by PGD with congenital disease can be donated for research and cultured to the blastocyst stage for hESC derivation. To date, disease mechanisms underlying several neurological disorders have been approached by using diseased-specific hESCs.

Fragile X syndrome (FXS) is one of the most common cognitive disorders. It is caused by the mutation of FMR1 gene, encoding FMRP protein [60]. The FMR1 gene contains CGG repeats at 5′ upstream of the promoter region, and healthy individual caries this region up to 55 repeats. CGG repeats could expand during gametogenesis, and when it reaches 200 repeats will lead to FMR1 gene hypermethylation and gene silencing. FXS-hESCs were derived from PGD blastocysts and showed normal properties of human pluripotent stem cells [61]. Noteworthy, although FXS-hESCs contain 200–1,000 CGG repeats, FMR1 gene is unmethylated and FMRP is expressed normally. The silence of FMRP protein is found upon the differentiation of FXS-hESCs. Abnormal neural differentiation process was found in FXS-hESCs, compared to normal control hESCs [62]. The defects of neuron derived from FXS-hESCs included neuronal morphology, timing of development, and the aberrant expression of key neural lineage markers [62]. In FXS-hESCs, the neural progenitor cells mainly give rise to GFAP-positive glial cells, while the control hESCs became Tuj1-positive neurons. In addition, FXS-hESC neurons reduced the frequency and amplitude of their action potential, as well as spontaneous synaptic activity.

Huntington's disease (HD) is a dominantly inherited neurodegenerative disorder caused by the expansion of a CAG repeat in HTT gene. HTT gene encodes an amino-terminal stretch of polyglutamines, called huntingtin protein (HTT). HD is characterized by motor, cognitive, and psychiatric abnormality, but the exact mechanisms show repeated HTT protein caused neuron degeneration is not yet clear. Notably, because of the exclusively monogenic character of HD, this enables the potential use of hESCs to model HD pathology and screen for drug candidates. Stable expression of mutant HTT protein was introduced into healthy hESCs [63]. Neurons derived from these hESC lines showed HTT aggregates and abundant cell death in the culture. This deleterious phenotype can be rescued by silencing mutant HTT expression [63]. Knockdown of another gene implicated in HD pathology, such as Rhes, was also shown to recover HD pathology in the mutant neurons [64]. HD-hESCs are able to recapitulate some of the dominant phenotypes found in animal models, permitting future study in a detailed human context.

Rett syndrome (RTT) is a monogenic X-linked neurodevelopmental disorder. Major RTT patients are affected by MeCP2 gene mutation and appear as autistic-like behavior, sensory defects, ataxia, and microcephaly. MeCP2 is a methyl CpG binding protein and acts as a global transcriptional repressor. By employing genome editing technology to introduce mutant MeCP2, isogenic RTT-hESCs were generated [65]. MeCP2-mutant neurons exhibited central molecular and cellular phenotypes of RTT, including morphology and physiological defects. Striking global gene expression was downregulated in MeCP2-mutant neurons, which reflected the significantly reduced protein synthesis and could be rescued by pharmacological and genetic manipulations [65]. Besides, the size of neuronal nuclei fails to enlarge at a normal rate during neural differentiation, compared to control hESCs [66]. This is accompanied by a significant reduction of ribonucleotide incorporation as well as the reduced level of brain-derived neurotrophic factor (BDNF). Reintroduction of MeCP2 could recover the nuclear size phenotype and BDNF expression level, suggesting the roles and functions of MeCP2 in RTT pathology [66].

Lesch-Nyhan syndrome (LNS) is a rare X-linked neurological disorder. Mutation of HPRT1 gene, encoding the hypoxanthine-guanine phosphoribosyl transferase (HGPRT), is a causative of LNS. HGPRT is an enzyme important for the generation of purine nucleotide, and the insufficiency of HGPRT leads to the accumulation of uric acid in the blood. Mental retardation is emerged as a symptom of LNS. There was an attempt to use hESCs to model LNS pathology. Mutant HGPRT was introduced into wild-type hESCs by homologous recombination [67]. LNS-hESCs resented several phenotypes mimicking LNS pathology, in particular uric acid accumulation. Although several downstream targets of HPRT1 mutations were explored, neural differentiation of LNS-hESCs has not yet been performed.

Malignant gliomas are the most aggressive nervous tumor found in both children and adults. Somatic mutation of H3F3A gene was found in major glioma patients. H3F3A gene encodes the histone H3 variant H3.3 and results in a Lys 27-to-methionine change (H3.3K27M) [68]; however, the role of H3.3K27M mutation in glioma formation is not fully understood. hESC lines carried H3.3K27K were generated and differentiated into neural progenitor cells [69]. Neural progenitor cells derived from H3.3K27M hESCs loss p53 expression and PDGFRA inactivation, leading to neoplastic transformation. Transcriptomic profiling reveals a resetting of the transformed neural progenitor cells to a developmentally more primitive stem cell state. This change is in accordance with major modifications of histone marks at numbers of master regulator genes [69]. The neural derivatives of these hESCs can also be used to screen for compounds that prevent tumor cell growth.

Down syndrome (DS) is caused by a trisomy 21 or extra chromosome 21, which is one of neurodevelopmental disorders manifested with cognitive abnormality. DS often associates with amyloid accumulation of early-onset Alzheimer disease (EOAD). This could be due to extra copies of over 400 genes that locate on chromosome 21. The critical region, 21q22.1–q22.3, contains genes, encoded amyloid protein, which is important in neurodevelopment and neurodevelopmental disorders [70]. As a result, accumulation of amyloid plaque in the brain leads to cognitive decline as EOAD in DS patients [71]. Previous report showed that hESCs were inhibited to differentiate into NPCs by accumulating of amyloid-*β* (A*β*1–42), which could be explained by the requirement of nonamyloidogenic pathway for hESCs to enter neural lineage [72].

Autism spectrum disorder (ASD) is another important neurodevelopmental disorder, manifested by aberration of social interaction and communication, as well as repetitive behaviors. While specific causes of autism spectrum disorders have yet to be found, many risk factors have been identified in the research literatures that may contribute to their development, including genetic factors. The deletion of 16p11.2 region on chromosome 16 is one of a well-studied ASD causative [73]. Recently, hESCs were reported in ASD model by genome engineering on 16p11.2 locus. Interestingly, transcription activator-like effector nuclease (TALEN), which is an essential tool in genomic editing, was capable of directed differentiating and highlighting hESCs as suitable model to study ASD pathology [74].

Based on the aforementioned examples, it is proved that hESCs present as a suitable platform system to model neurological disorders. Their application to the understanding of the molecular pathology of brain diseases can be significant in both basic research and therapeutic purposes.

## 7. Future Perspectives and Challenges

The notion of differentiation process of hESCs recapitulating the temporal changes found* in vivo* development has become widely accepted, not only for the nervous system, but also for other cellular lineages [13, 75]. Several early-onset neurological disorders showed the success of disease modeling by using hESCs. Immature phenotypes of neurons derived from hESCs hinder the applications of modeling for late-onset diseases [76]. Late-onset diseases could also be modeled by this system by progerin-induced aging [77]. Noteworthy, hESC differentiation system contains several limitations like other systems. Although the differentiation of hESCs displays an early stage of disease development, detailed characterization of* in vitro* neural derivatives is necessary in order to validate their* in vivo* counterparts and verify the stage of disease ontogeny.

In addition to hESCs, induced pluripotent stem cells (iPSCs) and induced neurons (iNs) have been intensively focused and employed as a disease modeling system [78, 79]. iPSCs and iNs could be generated from somatic cells of diseased-specific patients. Thus, these cells serve as a novel platform to functionally study specific mutations [78]. However, application of iPSCs and iNs to model diseases is restricted by several reasons, in particular epigenetic barriers of starting reprogrammed cells. Diseases which are related to imprinting genes and epigenetic anomaly, such as Fragile X syndrome [80], Angelman syndrome [81], and Prader-Willi syndrome [82], seem to be incompletely reprogrammed and unable to reset their epigenetic memory [32], which means iPSC and iN technology needed further development in order to overcome these issues [83].

Another challenge is the development of efficient protocol to derive specific neural derivatives. Each neurological disorder is usually affected by particular neuronal subtypes. Alternatively, the relevant neuronal subtypes are also needed to be isolated by using specific neuron reporter genes in order to obtain a pure population for further analysis. It is noted that the* in vitro* differentiation system cannot provide a spatial organization which exists as precise cell-specific microdomains or niche within the embryo. The further development of culture systems, combining with tissue engineering technology, will offer an improved microenvironment and increase differentiation efficiency of hESCs toward desired neuronal cell types. The use of hESC-derived neural derivatives to explore brain development and disease mechanisms is still in a developing phase, and when completed, this system will provide a tremendous promise for both scientists and clinicians.
